# Juvenile justice staff endorsement of HIV/STI prevention, testing, and treatment linkage

**DOI:** 10.1186/s40352-019-0096-7

**Published:** 2019-09-04

**Authors:** Sheena K. Gardner, Katherine S. Elkington, Danica K. Knight, Sofia Huang, Ralph J. DiClemente, Anne C. Spaulding, Carrie B. Oser, Angela A. Robertson, Connie Baird-Thomas

**Affiliations:** 1 Social Science Research Center, Mississippi State University, 1 Research Blvd., Suite 103, Starkville, MS 39759 USA; 2 Department of Psychiatry, Division of Child & Adolescent Psychiatry, Columbia University, 1051 Riverside Drive, #15, New York, NY 10032 USA; 3 Institute of Behavioral Research, Texas Christian University, 3034 Sandage Avenue, Fort Worth, TX 76109 USA; 4 College of Global Public Health, New York University, 715 #719, Broadway, New York, NY 10003 USA; 5 Rollins School of Public Health, Emory University, 1518 Clifton Road, Atlanta, GA 30322 USA; 6 Department of Sociology, University of Kentucky, 1531 Patterson Office Tower, Lexington, KY 40506 USA; 7 Social Science Research Center, Mississippi State University, 153 Mississippi Parkway, Canton, MS 39046 USA

**Keywords:** Juvenile justice, HIV, STI, Sexually transmitted infections, Staff attitudes, Prevention, HIV testing, HIV treatment, Linkage

## Abstract

**Background:**

While involvement in the legal system offers an opportunity to educate, screen, and treat high-risk youth, research shows that staff attitudes toward these practices can serve as barriers to implementation. The current study investigates the degree to which JJ staff endorse HIV prevention, testing, and treatment linkage practices with youth under community supervision and examines differences between individuals who supervise youth (e.g., juvenile probation officer) and those working in non-supervisory roles (e.g., case manager, assessment specialist).

**Methods:**

Juvenile justice staff consenting to participation in JJ-TRIALS completed an initial staff survey (*N* = 501). Survey items measured perceived importance of HIV/STI prevention (4 items); perceived importance of HIV/STI testing (7 items); and perceived importance of HIV/STI treatment linkage (8 items).

**Results:**

Confirmatory Factor Analysis (CFA) was computed (SAS CALIS procedure) for each of the three domains. Findings suggest that while staff recognize that youth are at risk for HIV/STIs and require provision of HIV/STI prevention and treatment linkage, attitudes concerning the importance of procuring or providing testing services for youth is substantially lower. Furthermore, analytic models comparing staff with and without supervision responsibilities (computed using SAS PROC MIXED) indicated that attitudes differed by site and staff responsible for supervision rated HIV treatment linkage practices as less important compared to non-supervising staff.

**Conclusions:**

Establishing partnerships with health agencies equipped with resources and skillsets to provide HIV/STI testing and related services may be an effective model to promote greater awareness and use of best practices among JJ staff and more effectively address the unmet needs of this high-risk population of youth.

## Background

Although persons under 25 years of age accounted for just over 40% of new HIV infections in the United States in 2016 (Centers for Disease Control and Prevention, 2017), between 67%–72% have never had an HIV test (Van Handel, Kann, Olsen, & Dietz, 2016) and approximately 60% of youth living with HIV are unaware of their status (Zanoni & Mayer, 2014). The Centers for Disease Control and Prevention (CDC) estimates that those unaware of their status account for about 40% of all new HIV infections (Gopalappa, Farnham, Chen, & Sansom, 2017).

Youth in the juvenile justice (JJ) system have disproportionately higher involvement in HIV risk behaviors such as unprotected sex and drug use (Belenko et al., 2008; Dembo, Belenko, Childs, Wareham, & Schmeidler, 2009; Elkington et al., 2008; Elkington, Bauermeister, & Zimmerman, 2010) and increased prevalence of both HIV and STIs (Gamarel et al., 2016). Furthermore, research documents a strong link between substance use and illegal activity (Ford & Rigg, 2015; Silva, Schrager, Kecojevic, & Lankenau, 2013) and between substance use and HIV/STI risk (Aalsma, Tong, Wiehe, & Tu, 2010; Teplin, Mericle, McClelland, & Abram, 2003). As youth under community supervision are frequently disconnected from services and may miss school-based or other community-located HIV/STI interventions, juvenile community supervision agencies are uniquely positioned to provide HIV/STI testing and prevention programming to a high-risk group of vulnerable youth (Elkington et al., 2015). Access to timely HIV prevention, including Pre-Exposure Prophylaxis (PrEP), can reduce risk of HIV infection among youth under community supervision, and early identification (i.e., HIV testing) with prompt referral to treatment for youth who are found to be living with HIV and STIs, may reduce the transmission of the infections in their communities (Allen, Gordon, Krakower, & Hsu, 2017; Donenberg, Emerson, & Kendall, 2018; Donenberg, Emerson, Mackesy-Amiti, & Udell, 2015; Godin et al., 2003; Tolou-Shams, Stewart, Fasciano, & Brown, 2010).

Best practices for identifying and addressing HIV can be conceptualized along a cascade of care (Gardner, McLees, Steiner, del Rio, & Burman, 2011; MacCarthy et al., 2015). The HIV Care Cascade includes identification and diagnosis (i.e., testing), linkage to medical care, receipt of antiretroviral therapy, and achieving viral suppression. While much of these activities may be outside the purview of the justice system, the first stage—testing—can occur in any setting (with involvement from medically trained personnel). Yet to implement screening in a systematic way, staff working within justice agencies must understand and embrace the practice, otherwise efforts will be unsuccessful or short-lived. A similar cascade of care model, the Behavioral Health (BH) Services Cascade (Belenko et al., 2017), has been used within JJ agencies as a framework for promoting knowledge of and improvement in substance use screening, referral, initiation and engagement in treatment (Knight et al., 2016). For the current study, the HIV Care and the BH Services Cascade frameworks have been adapted to reflect three broadly-defined HIV/STI-related services: prevention, testing, and treatment linkage. Prevention refers to activities involving education about safe sex practices, education on contracting HIV and STIs, and the influence that alcohol and other drugs can have on decisions around safe sex. Testing refers to biological detection of antibodies to the HIV virus and strategies for offering testing services (such opt-out screening; (Spaulding et al., 2015)). Treatment linkage refers to activities around connecting seropositive youth to appropriate treatment and following up with medical service providers to be sure services are received.

While involvement in the legal system offers an opportunity to educate, screen, and treat high-risk youth, research has also demonstrated the challenges of implementing HIV-related activities within justice settings (Braithwaite & Arriola, 2008; Draine et al., 2011; Meyer, Chen, & Springer, 2011). In general, a lack of staff endorsement for best practices can serve as a barrier to implementation. For instance, a study of JJ probation officers found that individuals who rated certain substance use service strategies as unimportant (e.g., routine, universal screening; use of screening results to inform decisions; use of active referral strategies such as scheduling appointments, providing transportation) were less likely to use those strategies with youth on their caseloads (Knight et al., 2019). Similarly, a lack of staff endorsement for HIV prevention, testing, and treatment linkage can serve as barriers to HIV service delivery. Surveys of staff belonging to justice, health, and community-based organizations working within adult correctional populations have identified how staff endorsement of HIV service practices affect service delivery in correctional facilities (Robillard et al., 2003; Sabharwal et al., 2010; Visher et al., 2014). A survey of jail-based health care workers revealed that while most felt it was important to conduct HIV tests and were confident recommending rapid HIV testing, smaller proportions of the staff reported confidence in providing test results to clients (Sabharwal et al., 2010). Staff responses to open-ended questions suggest the need for additional organizational support and individual training to improve HIV service delivery. Other research suggests that locales receiving support as they implement HIV services in their correctional facilities experienced improvements in staff perceptions of the acceptability and feasibility (Visher et al., 2014). To our knowledge, only one study included JJ staff attitudes towards HIV service provision. Robillard et al. (2003) found that frontline JJ staff believe the main responsibility of correctional institutions is maintaining security, not providing HIV-related services. The authors felt that JJ staff willingness to cooperate with healthcare workers providing HIV-related services in detention settings could be improved through education and training that would help address JJ staff’s misconceptions about HIV and improve understanding of the role they could play in HIV prevention, testing, and referral to treatment (Robillard et al., 2003). JJ staff only comprised 14% of the sample in Robillard et al.’s (2003) study, however, highlighting that current knowledge of JJ staff endorsement of components within the HIV care continuum would benefit from further investigation.

Previous research conducted in justice settings has identified line staff (e.g. probation officers, correctional officers) as key contributors to the (un)successful implementation of new practices (Rudes, Viglione, & Taxman, 2011). This knowledge, combined with findings that JJ staff believe the delivery of HIV-related services to extend beyond the scope of their position (Robillard et al., 2003), makes job responsibility an important factor to consider with respect to HIV service delivery in JJ settings. In a set of qualitative interviews with staff working in a correctional setting, researchers found that resistance against the introduction of HIV services varied, with staff responsible for delivering newly adopted services expressing more opposition (Robillard et al., 2003). When, for example, line staff struggle with reconciling past practices with new ones (Lin, 2002; Robillard et al., 2003) or perceive a lack of fairness in or exclusion from organizational decision-making concerning the implementation of new practices (Cox, 2013; Taxman & Gordon, 2009), they may be less likely to embrace implementation efforts.

The purpose of this study is to investigate the degree to which JJ staff endorse HIV prevention, testing, and treatment linkage practices with youth under community supervision and to examine differences between individuals who supervise youth (e.g., juvenile probation officer) versus those working in non-supervisory roles (e.g., case manager, assessment specialist). Because STIs are similar to HIV in contraction through sexual intercourse, and because JJ staff may be more aware of and comfortable with the provision of services for STI within JJ contexts, we have included STIs as part of the overall conceptualization and assessment.

## Methods

This study uses data collected as part of the National Institute on Drug Abuse (NIDA)-funded *Juvenile Justice-Translational Research on Interventions for Adolescents in the Legal System* (JJ-TRIALS) cooperative research initiative (Knight et al., 2016). JJ-TRIALS consists of six research centers (Columbia University, Emory University, Mississippi State University, Temple University, Texas Christian University, and University of Kentucky) and a coordinating center (Chestnut Health Systems). This research project was approved by the Institutional Review Boards of each participating research center. One of the primary goals of JJ-TRIALS is to reduce the unmet needs of justice-involved youth in the areas of substance use and HIV prevention. JJ-TRIALS aims to make systems level changes that encourage partnering JJ agencies and behavioral health (BH) organizations to improve substance use and HIV service delivery for affected youth. Through a series of structured activities, partner organizations were encouraged to examine services along the BH Services (Belenko et al., 2017), beginning with screening or need identification and progressing through linkage to appropriate care and to identify those areas in which “gaps” in service delivery occurred (Horan Fisher et al., 2018).

Each research center engaged six JJ agencies (e.g., county youth court or probation department) and at least one community-based BH service provider working with each JJ agency, resulting in 36 “sites” (a paired JJ and BH agency) in seven states (Florida, Georgia, Kentucky, Mississippi, New York, Pennsylvania, and Texas). Staff were recruited to participate in the study after attending an in-person orientation meeting in which all aspects of the study were explained and staff had an opportunity to ask questions. If staff were unable to attend the orientation, researchers contacted staff by phone to explain the study and interested individuals mailed consents to the research centers.

Beginning in August 2015, 739 (82%) of the eligible 904 leadership and line staff from JJ and BH agencies consented to participate in the JJ-TRIALS protocol and complete 4 staff surveys over a 2-year period. Participants had the choice to complete either a Qualtrics® web-based survey that used individualized email invitation links or a paper survey (Knight et al., 2019). The Time 1 survey response rate was 82%, yielding 607 completed surveys. Of these, 501 (83%) were JJ staff. Because the focus of this study is on HIV/STI service provision within JJ settings, only data collected from the 501 JJ staff are analyzed and reported.

### Measures

The focus of the current study is the staff of JJ agencies and endorsement of HIV/STI prevention, testing, and treatment linkage. Information was collected on the demographic characteristics of JJ staff (gender, age, race, and Hispanic ethnicity), years of experience working with youth, number of years with the current employer, position title, and job responsibilities. Respondents selected job responsibilities from a list of possible options (see Table 1). Those who indicated supervision of youth were coded as 1 “responsible for supervision;” all others were coded as 0 “not responsible for supervision.”

**Table 1:** Demographic Characteristics of Staff Survey Respondents

	*N*	%	Mean	SD
Gender				
Male	297	59		
Female	203	41		
Age			40.22	12.33
Years of Experience			14.94	8.69
Years with Current Employer			11.6	8.25
Race				
Black	122	25		
White	360	72		
Mixed	5	1		
Other	8	2		
Hispanic/Latino				
Yes	62	12		
No	437	88		
Job Level				
Probation Officer	299	60		
Supervisor	79	16		
Case Manager or Counselor	52	10		
Agency or Division Director	42	8		
Support/Other	22	44		
Missing	7	11		
Job Responsibilities				
Supervision of youth	322	64		
Case management	292	58		
Screening for substance use	232	46		
Comprehensive assessment	128	26		
Supervision/management of staff	120	24		
Aftercare or supportive services	115	23		
Substance use services	98	20		
HIV/STI services	9	2		
Other	48	10		

Items measuring endorsement of HIV/STI prevention, testing, and treatment linkage were developed specifically for JJ-TRIALS and were designed to map onto key elements of the HIV care cascade (Gardner et al., 2011) and BH services cascade. They were adapted from the Revised Recommendations for HIV Testing of Adults, Adolescents, and Pregnant women in Health-Care Settings (Centers for Disease Control and Prevention, 2006). The measure of *perceived importance of HIV/STI prevention* consisted of 4 items; *perceived importance of HIV/STI testing* consisted of 7 items; and *perceived importance of HIV/STI treatment linkage* consisted of 8 items. Some items referred to general practices across an agency (e.g., “2a-Recommending that all youth be tested for HIV as part of their service plan”), whereas others referred to specific practices used with individual youth (e.g., 3c-Following up with the service provider to be sure that the HIV positive youth is receiving HIV treatment). Respondents were asked to rate the importance of each item with response options ranging from “not important” (coded 1) to “very important” (coded 5). Scale scores were computed by obtaining the average of the scale items and multiplying by 10. “Percent important” was calculated by recoding responses of 4 or 5 (important, very important) as “important” (1) and responses of 1–3 (not important, slightly important, and moderately important) as “not important” (0). Table 2 displays the wording and descriptive statistics for each item.

**Table 2:** Staff Endorsement of HIV/STI Prevention Testing, and Treatment

Item Wording	Mean	SD	% Important
Importance of HIV/STI Prevention			
Informing youth about how being under the influence of alcohol or drugs can lead to risky sexual behaviors	4.19	0.97	82
Educating youth about safe sex practices	4.04	1.1	76
Providing information about how one gets an HIV infection and how one can transmit HIV to other people	3.97	1.15	72
Providing information about how one gets sexually transmitted infections	3.98	1.15	73
*Overall Prevention Score*^*a*^	*40.44*	*10.37*	
Importance of HIV/STI Testing			
Encouraging youth and their new partners to get tested for STIs before starting a sexual relationship	3.31	1.48	54
Encouraging youth and their new partners to get tested for HIV before starting a sexual relationship	3.29	1.47	53
Routinely screening all youth for HIV infection who are getting STI Tx	2.84	1.47	39
Using ‘opt-out’ HIV testing procedures	2.74	1.41	36
Using ‘opt-out’ STI testing procedures	2.75	1.41	36
Recommending that all youth be tested for other sexually transmitted infections (STIs) as part of their service plan	2.69	1.46	33
Recommending that all youth be tested for HIV as part of their service plan	2.59	1.44	31
*Overall Testing Score*^*a*^	*28.84*	*12.69*	
Importance of HIV/STI Tx			
Providing contact information - HIV Tx provider	3.77	1.39	69
Providing contact information - STI Tx provider	3.78	1.38	69
Promptly linking youth with HIV seropositive test results to HIV Tx services	3.8	1.41	68
Promptly linking youth with STI positive test results to STI Tx services	3.76	1.41	67
Following up with HIV positive youth to ensure they receive HIV Tx	3.62	1.45	63
Following up with STI positive youth to ensure they receive STI Tx	3.61	1.45	63
Following up with the service provider to be sure that the HIV positive youth is receiving HIV Tx	3.51	1.46	59
Following up with the service provider to be sure that the STI positive youth has received Tx	3.5	1.47	59
*Overall Treatment Score*^*a*^	*36.61*	*13.38*	

^a^Domain scores multiplied by 10

### Analysis plan

To determine whether items loaded onto the three conceptual domains as expected, Confirmatory Factor Analysis (CFA) was computed (SAS CALIS procedure) for each of the three domains: HIV/STI Prevention, Testing, and Treatment Linkage. CFA confirmed that all three measurement domains had single factor solutions. Item loadings for HIV/STI Prevention (items 1a-1d; see Table 2) ranged from .87 to .99, with a Cronbach’s alpha of .96. Item loadings for HIV/STI Testing (items 2a-2 g) ranged from .80 to .88, with a Cronbach’s alpha of .95. Item loadings for HIV/STI Treatment Linkage (items 3a-3 h) ranged from .79 to 1.00, with a Cronbach’s alpha of .98. Means, standard deviations, and percentage agreement (responses of 4 or 5 indicating “important” or “very important”) were calculated for each item and for each domain score.

Because staff respondents are nested within juvenile justice departments and individuals within the same workplace are likely to have similar attitudes, multilevel analysis (SAS PROC MIXED, Raudenbush, Bryk, & Congdon, 2005) was used to examine the relationship between job responsibility and importance of HIV/STI related practices, controlling for site membership. Three analytic models were computed, with each domain score as the dependent variable, job responsibility (Responsible for Supervision versus Not Responsible for Supervision) as the independent variable, and demographics (staff gender, Hispanic ethnicity and age) as covariates.

## Results

The characteristics of the respondents in the sample are displayed in Table 1. Respondents were primarily female (59%), White (72%), Probation Officers (60%), with job responsibilities pertaining to supervision (64%) and case management (58%). The mean age was 40 years (SD = 12.3), staff averaged 15 years of experience (SD = 8.7), and were employed over 11 years on average with their current agency (M = 11.6, SD = 8.25). Of note, while 20% of JJ staff reported that their job responsibilities included provision of substance use education and drug court (i.e. substance use services), only 2% (*n* = 9) reported responsibility for HIV/STI testing, treatment, and prevention services.

Table 2 presents means, SDs, and percent responding “important” on each item within the three HIV/STI domains. With respect to importance of HIV/STI prevention services, overall, staff reported that providing these services was “important” (m = 40.44, SD = 10.37). In particular, 82% of staff felt that providing information about how alcohol and drug use can lead to risky sexual behaviors was “important,” as was providing information on transmission of HIV and STIs and practicing safe sex (between 72%–76%). In contrast, overall, JJ staff perceived HIV/STI testing as slightly to moderately important (m = 28.84; SD = 12.69). While over half believed it was important to encourage JJ-involved youth to test for HIV (53%) and/or STIs (54%) at the beginning of a new relationship, between 31% and 39% viewed other related practices as important. For example, 39% thought routine HIV or STI testing was important while only about one-third felt that HIV (31%) or STI (33%) testing was important to include as part of the youth’s probation service plan. JJ staff’s rating of the importance of HIV testing practices was the lowest among the three domains. JJ staff perceived practices related to HIV/STI treatment as moderately important to important (m = 36.61, SD = 13.38). Nearly 70% reported that providing contact information for HIV and STI services (both 69%), and promptly linking youth infected with HIV or STIs to treatment (68% and 67%, respectively) was important. Of note, approximately 10% fewer (59%) felt it was important to follow-up with HIV or STI treatment providers to ensure that the seropositive youth on their caseload are receiving treatment.

Results of analyses comparing importance ratings based on job responsibilities (controlling for staff gender, Hispanic ethnicity and age) are located in Table 3. Results identified significant differences only on the Treatment Linkage domain. Specifically staff directly supervising youth reported lower agreement that providing HIV and STI treatment linkage was important (F_(1, 422)_ = 5.21; *p* = .0230). Although statistically non-significant, trends in the same direction were seen for Prevention. Differences in attitudes toward Testing were non-significant.

**Table 3:** HIV Domains by Staff Job Responsibility (supervision)

	Responsible for Supervision			Not Responsible for Supervision				
	N	Mean	SE	N	Mean	SE	*F*-value^a^	*P*-value
Prevention	315	40.50	0.98	170	42.25	1.13	3.45	0.0641
Testing	313	29.93	1.33	169	30.34	1.48	0.13	0.7226
Treatment	303	36.06	1.19	169	38.96	1.39	5.21	0.0230

^a^Adjusted for site, gender, ethnicity, and age

## Discussion

The current paper is among the first to explore endorsement of HIV/STI testing and related services among JJ community supervision staff. Findings suggest staff recognize that youth on their caseloads are at risk for HIV/STIs and require provision of HIV/STI prevention, treatment linkage, or both. However, JJ staff reported less agreement related to *their* responsibility in procuring or providing these services, particularly with respect to making HIV and STI testing a standard component of a youth’s supervision plan. Thus, there is a marked incongruence between JJ staff perceptions of the importance of HIV/STI prevention and testing, and the importance of their playing a role in the provision of these services, particularly testing within the JJ setting. Community supervision agencies can be an important source of HIV/STI prevention information and risk-reduction skills training. As in adult services, actual integration of HIV/STI prevention and testing services into the probation plan may be seen as off-mission to their job responsibilities (Robillard, Braithwaite, Gallito-Zaparaniuk, & Kennedy, 2011; Visher et al., 2014). Unlike provision of health services in a locked facility, provision of medical services for individuals under community supervision is not a constitutional right (see Estelle v. Gamble, 429 U.S. 97, 1976). In order to ensure that needs are met, it is essential to educate JJ staff about the importance of HIV/STI services and support the use of prevention, testing, and treatment linkage practices with JJ-involved youth who might not receive such public health intervention outside of the community supervision program.

Prior work has shown that conflicting job responsibilities of community supervision staff can be difficult to reconcile—both in enforcing legal requirements of supervision (the “law enforcement” role) and in assisting the individual in successful community adjustment (the “rehabilitative” or “social worker” role; Clear & Latessa, 1993). Difficulty prioritizing which role to emphasize can influence job performance, and combined with high levels of occupational stress (White, Aalsma, Holloway, Adams, & Salyers, 2015), can lead staff to focus primarily on the “security and control” mission of juvenile supervision (Robillard et al., 2011; Rudes et al., 2011). Correspondingly, we found that staff providing direct supervision to youth reported significantly lower mean scores overall regarding the importance of delivering HIV/STI treatment linkage than did JJ staff not responsible for direct supervision. Such perceptions may affect delivery of HIV/STI testing in these settings, shown to be less than 1% in a national survey of juvenile community supervision agencies (Elkington et al., 2018).

Given the incongruence between JJ staff’s recognition of the need for HIV/STI services and their willingness to provide services, additional or ancillary staff may be needed to ensure provision of HIV/STI prevention information and risk-reduction training as well as HIV/STI screening. The additional cost associated with new and specified staff is a key consideration. In the cost-constrained environment in which most of these systems exist, finding additional sources of revenue specifically targeted for HIV/STI prevention and screening is challenging. As an alternative, it may be feasible to link another key public system, local public health departments, to conduct the HIV/STI prevention and testing for JJ-involved youth. Public health departments are charged with providing both of these services, employ highly skilled personnel who have expertise in working with vulnerable youth, and may be able to provide an efficient and effective pathway to additional health department services for justice-involved youth. While this latter alternative is attractive, there are few studies that have examined whether effective relationships between community supervision agencies and local health departments can be developed and sustained.

### Limitations

We should note a number of limitations of this study. The study sample of 36 JJ sites in seven states was non-random. Therefore, despite structural and demographic diversity in this sample, the degree of generalizability across other states or within other counties or agencies in the states represented is unknown. It is also unknown whether individuals who chose not to respond to the survey differed from respondents in their perceptions of the importance of activities related to the HIV care continuum. Additionally, these data represent self-reported measures of the perceived importance of HIV/STI prevention, testing, and treatment linkage. It is difficult to determine whether attitudes toward best practices are correlated with actual behaviors related to the HIV care continuum (e.g., delivery of prevention, testing, or treatment linkage).

## Conclusions

Prior work has highlighted the difficulty of improving the delivery of HIV-related services within justice settings (Pearson et al., 2014; Robillard et al., 2011; Visher et al., 2014), which suggests that systems-level interventions are needed to change practice (Taxman & Belenko, 2012). Establishing partnerships with health agencies that are equipped with resources and skillsets to provide HIV and STI testing and related services may be an effective model to promote greater awareness and use of best practices for HIV and STI among JJ staff and more effectively address the unmet needs of this high-risk population of youth. While there is a lack of literature related to evidence-based strategies aimed at increasing service system collaboration between JJ agencies and partnering service agencies, successful partnerships have been documented in adult settings (Belenko et al., 2013; Pearson et al., 2014; Visher et al., 2014) and suggest such an approach may be an effective way forward in JJ agencies and a significant step toward reducing individual youth and public health risk.

## Data Availability

Not applicable.

## Abbreviations

BH: Behavioral Health
HIV: Human Immunodeficiency Virus
JJ: Juvenile Justice
JJ-TRIALS: Juvenile Justice-Translational Research on Interventions for Adolescents in the Legal System
NIDA: National Institute on Drug Abuse
PrEP: Pre-Exposure Prophylaxis
STI: Sexually Transmitted Infection
